# THE ESTABLISHMENT OF A COMMUNITY NETWORK TO SUPPORT FAMILY CAREGIVERS

**DOI:** 10.1093/geroni/igac059.1365

**Published:** 2022-12-20

**Authors:** Tsuann Kuo

**Affiliations:** Chung Shan Medical University, South District, Taichung, Taiwan (Republic of China)

## Abstract

In 2021, 114 Caregiver Resource Sites were established in Taiwan to provide case management and services for family caregivers who may experience caregiving burnout due to long-term care. Each Caregiver Resource Site is responsible for serving 60-100 caregivers. An effort was made to recruit volunteers and gather resources so caregivers can be surrounded in the care-friendly community network, including care counseling, support groups, caregiving skill classes, relaxation activities, caregiver café, and in-home coaching for hard-to-care moments. By forming this community network to support caregivers, it has reduced caregiver burnout and avoided possibly negative tragedies due to caregiving.

